# Non-contrast computed tomography features predict intraventricular hemorrhage growth

**DOI:** 10.1007/s00330-023-09707-9

**Published:** 2023-05-22

**Authors:** Jawed Nawabi, Frieder Schlunk, Andrea Dell’Orco, Sarah Elsayed, Federico Mazzacane, Dmitriy Desser, Ly Vu, Estelle Vogt, Haoyin Cao, Maik F. H. Böhmer, Burak Han Akkurt, Peter B. Sporns, Marco Pasi, Ulf Jensen-Kondering, Gabriel Broocks, Tobias Penzkofer, Jens Fiehler, Alessandro Padovani, Uta Hanning, Andrea Morotti

**Affiliations:** 1 Department of Radiology, Charité - Universitätsmedizin Berlin, Campus Mitte, Humboldt-Universität Zu Berlin, Freie Universität Berlin, Berlin Institute of Health, Charitéplatz 1, 10117 Berlin, Germany; 2 Berlin Institute of Health (BIH), BIH Biomedical Innovation Academy, Berlin, Germany; 3 Department of Neuroradiology (CCM), Charité - Universitätsmedizin Berlin, Campus Mitte, Humboldt-Universität Zu Berlin, Freie Universität Berlin, Berlin Institute of Health, Berlin, Germany; 4 Department of Diagnostic and Interventional Neuroradiology, University Medical Center Hamburg Eppendorf, Hamburg, Germany; 5 Department of Brain and Behavioral Sciences, University of Pavia, Pavia, Italy; 6 U.C. Malattie Cerebrovascolari E Stroke Unit, IRCCS Fondazione Mondino, Pavia, Italy; 7 Department of Radiology, University Hospital Muenster, Muenster, Germany; 8 Department of Neuroradiology, Clinic for Radiology and Nuclear Medicine, University Hospital Basel, Basel, Switzerland; 9 Department of Neurology, University Hospital of Tours, Tours, France; 10 Department of Neuroradiology, University Hospital Schleswig-Holstein, Campus Lübeck, Lübeck, Germany; 11 Department of Radiology, Charité - Universitätsmedizin Berlin, Campus Virchow Klinikum, Humboldt-Universität Zu Berlin, Freie Universität Berlin, Berlin Institute of Health, Berlin, Germany; 12 Department of Clinical and Experimental Sciences, Neurology Clinic, University of Brescia, Brescia, Italy; 13 Neurology Unit, Department of Neurological Sciences and Vision, ASST-Spedali Civili, Brescia, Italy

**Keywords:** Stroke, Cerebral hemorrhage, Tomography, Prognosis

## Abstract

**Objectives:**

Non-contrast computed tomography (NCCT) markers are robust predictors of parenchymal hematoma expansion in intracerebral hemorrhage (ICH). We investigated whether NCCT features can also identify ICH patients at risk of intraventricular hemorrhage (IVH) growth.

**Methods:**

Patients with acute spontaneous ICH admitted at four tertiary centers in Germany and Italy were retrospectively included from January 2017 to June 2020. NCCT markers were rated by two investigators for heterogeneous density, hypodensity, black hole sign, swirl sign, blend sign, fluid level, island sign, satellite sign, and irregular shape. ICH and IVH volumes were semi-manually segmented. IVH growth was defined as IVH expansion > 1 mL (eIVH) or any delayed IVH (dIVH) on follow-up imaging. Predictors of eIVH and dIVH were explored with multivariable logistic regression. Hypothesized moderators and mediators were independently assessed in PROCESS macro models.

**Results:**

A total of 731 patients were included, of whom 185 (25.31%) suffered from IVH growth, 130 (17.78%) had eIVH, and 55 (7.52%) had dIVH. Irregular shape was significantly associated with IVH growth (OR 1.68; 95%CI [1.16–2.44]; *p* = 0.006). In the subgroup analysis stratified by the IVH growth type, hypodensities were significantly associated with eIVH (OR 2.06; 95%CI [1.48–2.64]; *p* = 0.015), whereas irregular shape (OR 2.72; 95%CI [1.91–3.53]; *p* = 0.016) in dIVH. The association between NCCT markers and IVH growth was not mediated by parenchymal hematoma expansion.

**Conclusions:**

NCCT features identified ICH patients at a high risk of IVH growth. Our findings suggest the possibility to stratify the risk of IVH growth with baseline NCCT and might inform ongoing and future studies.

**Clinical relevance statement:**

Non-contrast CT features identified ICH patients at a high risk of intraventricular hemorrhage growth with subtype-specific differences. Our findings may assist in the risk stratification of intraventricular hemorrhage growth with baseline CT and might inform ongoing and future clinical studies.

**Key Points:**

*• NCCT features identified ICH patients at a high risk of IVH growth with subtype-specific differences.*

*• The effect of NCCT features was not moderated by time and location or indirectly mediated by hematoma expansion.*

*• Our findings may assist in the risk stratification of IVH growth with baseline NCCT and might inform ongoing and future studies.*

**Supplementary Information:**

The online version contains supplementary material available at 10.1007/s00330-023-09707-9.

## Introduction

More than 5.5 million patients suffer from intracerebral hemorrhage (ICH) annually [1] and ICH represents the most severe form of stroke, with mortality and severe disability rates approaching 50% at 1 month and exceeding 75% at 1 year, respectively [2, 3]. Therapeutic strategies have failed to show overall beneficial treatment effects in previous ICH trials over the past years. Therefore, new candidates for potential treatment strategies are holding vast research attention. Intraventricular extension of intracerebral hemorrhage (IVH) occurs in 40% of ICH patients and is a well-established predictor of poor functional outcome [4]. IVH is a dynamic and potentially modifiable process (IVH growth) [5, 6] which can present either as interval increases in IVH volume (expansion IVH, eIVH) or as a delayed development of IVH (dIVH) on subsequent neuroimaging [7, 8]. Furthermore, both have shown a strong relationship with a poor functional outcome [5, 6] and hold potential to improve outcome prediction models for clinical ICH management [9]. Recent evidence suggested that non-contrast computed tomography (NCCT) features might help identify patients at risk of IVH growth; however, the exact relationship with eIVH and dIVH remains unclear [10]. Therefore, we hypothesized that both subgroups are independently predicted by distinct NCCT features. Recognizing that eIVH and dIVH vary in terms of chronological order and ICH location on the one hand, and on the other hand are common phenomena in patients with acute HE, we additionally hypothesized a potential link with the effects exerted by the NCCT features [8]. To test and evaluate our hypotheses, we performed a three-fold approach: Firstly, we tested and validated the association of NCCT features and IVH growth in an independent multicenter cohort. Secondly, we conducted two subgroup analyses to identify differences in NCCT markers independently associated with eIVH and dIVH. Thirdly, we evaluated if the effects were mediated by acute HE and moderated by ICH location and time.

## Material and methods

This multicenter retrospective study was approved by the ethics committee (Charité Berlin, Germany [protocol number EA1/035/20], University Medical-Center Hamburg, Germany [protocol number WF-054/19], University Hospital Muenster, Germany [protocol number 2017–233-f-S], and IRCCS Mondino Foundation, Pavia, Italy [protocol number 20190099462]), and written informed consent was waived by the institutional review boards. All study protocols and procedures were conducted in accordance with the Declaration of Helsinki. Patient consent was not needed due to the retrospective nature of the study.

### Study population

**Fig. 1:**
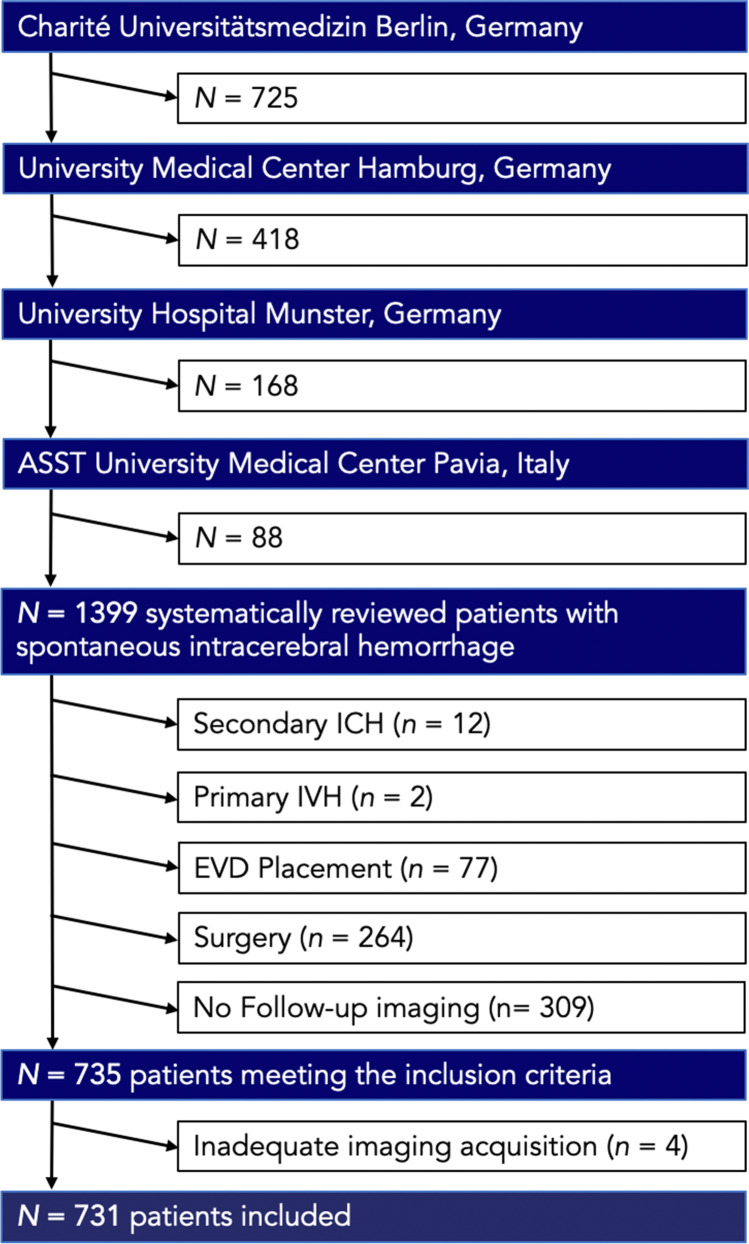
Patient flowchart. ICH, intracerebral hemorrhage; IVH, intraventricular hemorrhage; EVD, extraventricular drainage

We retrospectively selected ICH patients admitted at four tertiary stroke centers in Germany and Italy (Charité University Hospital, Berlin, Germany (2015–2019); University Medical Center Hamburg-Eppendorf (2015–2019), Germany; University Hospital Muenster (2011–2015), Germany; and IRCCS Mondino Foundation, Pavia, Italy (2017–2019)). Patients were selected according to the following inclusion criteria: (1) primary, spontaneous ICH, (2) age > 18 years, (3) baseline NCCT images acquired within 24 h from onset/last seen well (LSW) with or without CT angiography (CTA); patients with (1) secondary ICH, (2) missing follow-up imaging or (3) follow-up NCCT performed after 72 h, and (4) surgical procedures including craniotomy and placement of external ventricular drain (EVD). A patients’ selection flowchart is provided in Fig. 1.

### Clinical variables

Clinical data obtained from medical records included age, sex, history of hypertension and diabetes mellitus, systolic blood pressure, anticoagulation and antiplatelet treatment, Glasgow Coma Scale (GCS) at admission, time from symptom onset/LSW to imaging, and modified Rankin Scale (mRS) at 90 days.

### Image analysis

NCCT images were acquired based on local CT protocols at each participating site. Imaging data were retrieved in Digital Imaging and Communications in Medicine (DICOM) format from the local picture archiving and communication system (PACS) servers and anonymized in compliance with the local guidelines. DICOM data were transformed into Neuroimaging Informatics Technology Initiative (NifTI) format and were independently reviewed by two stroke imaging experienced readers (Berlin: J.N. and L.V.; Hamburg: J.N. and S.E.; Muenster: J.N. and S.E.; Pavia: J.N. and F.M.). All readers independently reviewed images in a random order, were blinded to all demographic and outcome data, and were not directly involved in the clinical care of the enrolled patients. Images were analyzed for the presence of IVH and ICH location. Supratentorial bleedings in cortical and subcortical locations were classified as lobar whether hemorrhages involving the thalamus, basal ganglia, internal capsule, and deep periventricular white matter were classified as deep [11]. Brainstem and cerebellar bleedings were classified as infratentorial [12].Volume quantifications of ICH and IVH were performed on NCCT images with semimanual planimetric measurements. The region of interest (ROI) histogram for ICH and IVH segmentation was sampled between 20 and 80 Hounsfield units (HU) to exclude voxels that likely belonged to the cerebrospinal fluid or calcification. ROIs were delineated using the Analyze 11.0 Software and ITK-SNAP 3.8.0 Software (Penn Image Computing and Science Laboratory, 2019) [13, 14]. All NCCT markers were rated on axial NCCT images by two raters (J.N. with 6 years of experience in ICH imaging research and L.V. with 3 years of experience in ICH research) to determine the following nine markers as previously reported [15]: (1) irregular shape, (2) satellite sign, (3) island sign which were characterized as markers of shape [16]. Markers of density consisted of (1) heterogeneous density, (2) swirl sign, (3) black hole sign, (4) blend sign, (5) fluid sign, and (6) hypodensities [16]. All markers were rated inconsideration of the consensus definitions proposed by the International NCCT ICH Study Group [16]: In brief, irregular shape was defined as foci of hematoma margin irregularities in the largest hematoma region according to the Barras shape scales I–V [16, 17]. The presence of irregular shape was defined after dichotomization with a Barras scale of ≥ III [16]. Satellite sign was defined as a hematoma separate from the main hematoma (1–20 mm distance) with a maximum diameter of 10 mm [16, 18]. The island sign consisted of at least three scattered small hematomas all separate from the main hematoma or at least four small hematomas, some or all of which may connect with the main hematoma [16, 19]. Heterogeneous density was evaluated as foci of hypoattenuation in within the largest hematoma compared to the brain parenchyma according to the Barras density scales I–V [16, 17]. The presence of heterogeneous density was defined after dichotomization with a Barras scale of ≥ III [16]. The swirl sign was defined as a region of hypo- or isoattenuation compared with the brain parenchyma. The region may be rounded, streak-like, or irregular and does not require a strict encapsulation within the hematoma [20, 21]. The black hole sign consisted of a relatively hypodense area which is encapsulated within a hyperdense area and which is not connected with the adjacent brain tissue [21, 22]. The relatively hypodense area has an identifiable border and a difference of at least 28 HU between the two density regions [22, 23]. Blend sign was defined as a hypoattenuating area adjacent to a hyperattenuating area of the hematoma, with a clear separation between them at a density difference of at least 18 Hounsfield Units (HU) [23, 24]. Fluid sign referred similarly to the presence of one distinct hypoattenuating area above and one hyperattenuating area below a discrete straight line of separation, yet irrespective of its density measurements [16, 25]. The imaging sign hypodensities were defined as any hypodense region strictly encapsulated within the hemorrhage with any shape, size, and density which does not require a density measurement [16, 26]. Illustrative examples on NCCT marker ratings are provided in Supplementary Figure 1. A subset of patients (*n* = 100) was randomly selected and presented again for a second reading to one rater (J.N. with 6 years of experience in ICH imaging research). Images for the second reading were presented in a random order 3 months later to minimize the recall of the images.

### Outcomes

The main outcome of the analysis was the occurrence of any IVH growth. In secondary analysis, the same regression models were repeatedly stratified by the IVH growth type, distinguishing eIVH from dIVH.

### Statistical analysis

Data were tested for normality and homogeneity of variance using histogram plots and the Shapiro–Wilk test. Descriptive statistics are presented as counts (percentages [%]) for categorical variables and compared with the *χ*^2^ test, mean (standard deviation [SD]) for continuous normally distributed variables, and medians (interquartile range [IQR]) for non-normal continuous variables and compared with the Mann–Whitney test, respectively. Interrater agreement for ICH and IVH volume quantifications was calculated and expressed as intraclass correlation coefficient (ICC) with 95% upper and lower confidence intervals (CI) from pairs of two readers (E.V. and L.V.) [27, 28]. Interrater and intrarater agreements for the readings of NCCT markers were calculated and expressed as Cohen’s *κ* statistic with 95% upper and lower CI [29].

### Logistic regression analysis

Multivariate regression analysis was performed to identify independent variables associated with eIVH and dIVH in patients with IVH growth. Candidate variables were selected based on prior knowledge of clinical significance and sample size for the following: age, sex, hypertension, admission GCS, use of oral anticoagulation, and supratentorial ICH location [30]. Collinear covariates, as expressed by a variance inflation factor (VIF) of greater than 3, were identified and removed from the model if necessary [31]. Variables were fitted together using backward elimination with a *p* value criterion of less than 0.05 [32]. Given for selected variables are odds ratios (OR) with 95%CI and corresponding beta coefficients. For visual display, adjusted beta coefficients of significant independent variables were plotted. A statistically significant difference was set at a *p* value of less than 0.05.

### Moderation and mediation analysis

Time [8, 33] and location [34] were hypothesized to moderate the effect of the NCCT markers on IVH growth and subgroups (eIVH and dIVH). The PROCESS SPSS macro version 2.13 model 2 for moderation analysis was used to calculate the regression coefficients for the respective NCCT marker in each group (IVH growth, eIVH, dIVH) independently (Supplementary Material) [35]. Hematoma expansion (defined as continuous growth > 6 mL or relative growth > 33%) hypothesized to mediate the effect of NCCT markers on IVH growth and subgroups was assessed in a mediation model (Supplementary Material). The PROCESS SPSS macro version 2.13 model 4 for mediation analysis was used to calculate three pathways (Supplementary Material) [36]. Pathway a determined the regression coefficients for the effect of the NCCT marker on the mediator, pathway b examined the association between the mediator and IVH growth or subgroups, and pathway c estimated the total and direct effect of the NCCT marker on IVH growth and subgroups, respectively. Pathway ab calculated the indirect intervention effects. To test the significance of the indirect effect, the macro generated bias-corrected bootstrapped 95%CI [36]. Significant mediation was determined if the CI around the indirect effect did not include zero [36]. Analyses were performed using the statistical software package SPSS version 25® (IBM Corporation, 2019) and R Statistics® Version 3.5.1 (R Core Team. R: A Language and Environment for Statistical Computing. R Foundation for Statistical Computing, 2018).

### Supplementary analysis

A second multivariate regression analysis was performed to identify the effect of eIVH and dIVH on the functional outcome (Supplementary Table 4). A receiver operating curve (ROC) analysis was performed to evaluate and compare the discriminatory accuracies of IVH growth, HE, and revised HE in the prediction of functional outcome and mortality (Supplementary Material Table 5).

### Data availability statement

The datasets that support the findings of our study are available upon reasonable request from the corresponding author; however, prior approval of proposals may apply by our institution’s data security management and a signed data sharing agreement will then be approved.

## Results

A total of 731 out of 1399 patients met the inclusion criteria, as shown in Fig. 1, and 185 (25.31%) patients suffered from IVH growth, of whom 130 (17.78%) had an eIVH and 55 (7.52%) a dIVH. Patients with IVH growth had a lower admission GCS, shorter time from symptom onset/LSW to imaging, smaller hematoma volumes on both admission and follow-up imaging, and a higher frequency of hematomas in the basal ganglia. Within the IVH growth subgroups, patients with eIVH also had more deep bleedings, in contrast to dIVH that showed with higher frequencies of lobar hematomas. IVH volumes were higher in patients with eIVH compared to those in patients with dIVH on follow-up imaging. A detailed summary of the study population’s characteristic is provided in Table 1. Interobserver agreement for both ICH and IVH segmentations was excellent, as presented in the supplementary material. Interrater agreements for the NCCT features were substantial to excellent (*n* = 731; Cohen’s *κ* from 0.74 to 0.95) with good to excellent intrarater agreements (*n* = 100; Cohen’s *κ* from 0.81 to 0.98), as presented in the supplementary material. The presence of irregular shape was associated with 1.68 higher odds of IVH growth after adjustment for potential confounders in multivariable logistic regression (Table 2). In the subgroup analysis, irregular shape remained independently associated with an almost three times higher risk for dIVH (OR 2.72; 95%CI 1.91–3.33; *p* value 0.016), as shown in Table 3, whereas hypodensities were associated with increased odds of eIVH (OR 2.06; 95%CI 1.48–2.64; *p* value 0.0015). Finally, the moderator analysis demonstrated that the two interaction terms (Int. 1: NCCT marker × time; Int. 2: NCCT marker × location) had no significant effect on the direct association between the respective NCCT marker and IVH growth. The effect of the respective NCCT marker on IVH growth and its subtypes was not indirectly mediated by HE. Detailed results are presented in the supplementary material.

**Table 1:** Baseline demographic and clinical characteristics by patients with intraventricular hemorrhage growth (IVH growth) associated with intracerebral hemorrhage and no IVH growth (no IVH growth)

	All (*n* = 731)	No IVH growth (*n* = 546)	IVH growth (*n* = 185)	*p*	Expansion IVH (*n* = 130)	Delayed IVH (*n* = 55)	*p*
Age (years), median (IQR)	73 (62–80)	73 (61–78)	74 (63–81)	0.701	79 (60.8)	37 (67.3)	0.346
Female, *n* (%)	318 (43.5)	297 (54.4)	116 (62.7)	0.05	79 (60.8)	37 (67.3)	0.403
Hypertension, *n* (%)	613 (83.9)	463 (84.8)	150 (81.1)	0.391	106 (81.5)	44 (80)	0.807
Diabetes mellitus, *n* (%)	125 (17.1)	98 (17.9)	27 (14.6)	0.483	19 (14.6)	8 (14.5)	0.99
SPB (mmHg), median (IQR)	165 (145–195)	166 (145–194)	170 (147.5–200)	0.244	169 (147–200)	173 (147.5–212)	0.714
Anticoagulation, *n* (%)	193 (26.4)	137 (25.1)	56 (30.3)	0.155	40 (30.8)	16 (29.1)	0.796
Antiplatelet, *n* (%)	335 (45.8)	243 (44.5)	92 (49.7)	0.598	64 (49.2)	28 (50.9)	0.835
GCS, median (IQR)	13 (8–15)	11 (4–14)	11 (6–14)	< 0.001	11 (6–14)	12 (7–15)	0.129
Δ symptom onset to imaging (hours), median (IQR)	4.39 (1.8–15.09)	5.2 (1.85–17.3)	3.53 (1.36–13.72)	0.009	4.47 (1.4–14.38)	1.93 (1.25–12.29)	0.124
ICH volume on admission (mL), median (IQR)	16.12 (6.15–35.85)	24.13 (10.94–53.59)	14.58 (5.88–32.21)	< 0.001	26.27 (8.43–47.51)	24.44 (11.1–53.7)	0.525
ICH volume on follow-up (mL), median (IQR)	17.52 (6.74–41.47)	30.31 (11.53–77.08)	14.13 (6.13–34.27)	< 0.001	36.25 (22.9–84.41)	34.65 (11.15–73.96)	0.177
IVH volume on admission (mL), median (IQR)	0 (0–7.96)	0 (0–5.25)	2.10 (0–14.68)	< 0.001	8.03 (1.94–26.06)	0	-
IVH volume follow-up, (mL), median (IQR)	0.28 (0–9.17)	0 (0–3.47)	12.31 (3.6–39.63)	< 0.001	17.95 (7.75–50.28)	3.11 (0.9–19.25)	< 0.0001
IVH on admission, *n* (%)	331 (45.3)	201 (36.8)	130 (70.3)	< 0.001	130 (100)	0 (0)	-
IVH on follow-up, *n* (%)	382 (52.3)	200 (36.6)	182 (98.4)	< 0.001	130 (100)	55 (100)	-
Hematoma and IVH growth							
HE, *n* (%)	162 (22.2)	90 (12.31)	72 (38.27)	< 0.001	49 (37.7)	23 (41.8)	0.599
gIVH, *n* (%)	18 (25.31)	0	185 (100)	< 0.001	130 (100)	55 (100)	-
Revised HE, *n* (%)	273 (37.3)	88 (16.1)	185 (100)	< 0.001	130 (100)	55 (100)	-
NCCT marker							
IRR shape	388 (53.08)	266 (48.72)	122 (65.95)	< 0.001	84 (64.62)	38 (69.09)	0.481
Satellite sign, *n* (%)	284 (38.9)	202 (37.0)	82 (44.3)	0.182	29 (22.3)	10 (18.2)	0.529
Island sign, *n* (%)	100 (13.7)	66 (12.1)	34 (18.4)	0.085	13 (10)	5 (9.1)	0.849
HET density	121 (16.55)	78 (14.29)	43 (23.24)	0.266	29 (22.31)	14 (25.4)	0.402
Swirl sign, *n* (%)	464 (63.5)	328 (60.1)	136 (73.5)	0.001	115 (88.5)	46 (83.6)	0.372
BHS, *n* (%)	174 (23.8)	121 (22.2)	53 (28.6)	0.073	14 (10.8)	11 (20)	0.093
Blend sign, *n* (%)	88 (12.0)	68 (12.5)	20 (10.8)	0.553	3 (2.3)	5 (9.1)	0.038
Fluid sign, *n* (%)	56 (7.7)	44 (8.1)	12.0 (6.5)	0.487	3 (2.3)	5 (9.1)	0.038
Location							
Supratentorial, *n* (%)	620 (84.82)	481 (88.1)	165 (89.19)	0.567	117 (90.0)	48 (87.27)	0.363
Lobar, *n* (%)	280 (38.36)	214 (39.2)	65 (35.1)	0.445	37 (28.5)	28 (50.9)	0.003
Basal ganglia, *n* (%)	292 (39.95)	208 (38.1)	84 (45.4)	0.023	66 (50.8)	18 (32.7)	0.006
Thalamic, *n* (%)	48 (6.66)	32 (5.9)	16 (8.6)	0.094	14 (10.8)	2 (3.6)	0.016
Brainstem, *n* (%)	44 (6.02)	38 (7.0)	6 (3.2)	0.206	5 (3.8)	1 (1.8)	0.465
Cerebellar, *n* (%)	67 (9.2)	53 (9.7)	14 (7.6)	0.206	8 (6.2)	6 (10.9)	0.985
Clinical outcome							
mRS 0–3, *n* (%)	187 (25.58)	145 (26.6)	27 (14.59)	< 0.001	18 (13.85)	9 (16.36)	0.324
mRS 4–6, *n* (%)	544 (74.41)	365 (66.8)	158 (85.41)	< 0.001	112 (86.2)	46 (83.64)	0.487

*BHS*, black hole sign; *NCCT markers*, non-contrast computed tomography markers; *HE*, hematoma expansion; *eIVH*, expansion; *FU*, follow-up; *gIVH*, IVH growth; *HET density*, heterogeneous density; *ICH*, intracerebral hemorrhage; *IQR*, interquartile range; *IRR shape*, irregular shape; *IVH*, intraventricular hemorrhage; *GCS*, Glasgow Coma Scale; *EED*, edema extension distance; *mRS*, modified Rankin Scale; *p*, *p* value; *SBP*, systolic blood pressure

**Table 2:** Multivariate logistic regression analysis of predictors of intraventricular hemorrhage (IVH) growth

	IVH growth		
	OR (95%CI)	*β*	*p*
Age	1.01 (1.0–1.03)	0.01	0.055
Gender (ref: female)	1.50 (1.04–2.17)	0.4	0.032
Hypertension (ref: no)	0.79 (0.49–1.28)	− 0.23	0.343
Anticoagulation (ref: no)	1.14 (0.76–1.72)	0.13	0.52
Admission GCS	0.90 (0.87–0.94)	− 0.1	< 0.001
Supratentorial (ref: no)	0.55 (0.38–0.8)	− 0.601	0.002
IRR Shape (ref: no)	1.68 (1.16–2.44)	0.52	0.006
Satellite sign (ref: no)	0.79 (0.53–1.19)	− 0.23	0.264
Island sign (ref: no)	1.08 (0.73–1.59)	0.08	0.698
HET density (ref: no)	1.51 (0.96–2.37)	0.41	0.072
Swirl sign (ref: no)	1.20 (0.76–1.90)	0.18	0.442
Black hole sign (ref: no)	0.97 (0.56–1.69)	− 0.03	0.914
Blend sign (ref: no)	0.78 (0.44–1.37)	− 0.25	0.383
Fluid sign (ref: no)	0.58 (0.29–1.17)	− 0.54	0.127
Hypodensities (ref: no)	1.25 (0.83–1.88)	0.22	0.289

*β*, beta regression coefficient; *CI*, confidence interval; *GCS*, Glasgow Coma Scale; *HET density*, heterogeneous density; *IVH*, intraventricular hemorrhage; *IRR shape*, irregular shape; *OR*, odds ratio; *p*, *p* value; *ref*, reference

**Table 3:** Multivariate logistic regression analysis of predictors of intraventricular hemorrhage (IVH) growth given separately for patients with IVH expansion and delayed IVH

	IVH expansion			Delayed IVH		
	OR (95%CI)	*β*	*p*	OR (95%CI)	*β*	*p*
Age	1.0 (0.98–1.02)	− 0.0001	0.991	1.05 (1.02–1.09)	0.05	0.001
Gender (ref: female)	1.60 (1.06–2.14)	0.47	0.088	2.51 (1.79–3.21)	0.92	0.011
Hypertension (ref: no)	0.95 (0.28–1.62)	− 0.05	0.875	0.68 (− 0.19–1.55)	− 0.38	0.39
Anticoagulation (ref: no)	1.13 (0.59–1.67)	0.12	0.656	0.88 (0.12–1.64)	− 0.13	0.734
Admission GCS	1.02 (0.97–1.08)	0.02	0.430	0.88 (0.80–0.96)	− 0.13	0.0013
Supratentorial (ref: no)	0.60 (0.06–1.14)	− 0.51	0.060	0.56 (− 0.14–1.26)	− 0.58	0.104
IRR shape (ref: no)	1.05 (0.46–1.63)	0.05	0.876	2.72 (1.91–3.53)	1.0	0.016
Satellite sign (ref: no)	0.72 (0.06–1.38)	− 0.33	0.332	0.55 (− 0.43–1.53)	− 0.59	0.236
Island sign (ref: no)	0.87 (0.06–1.67)	− 0.15	0.726	0.94 (− 0.20–2.08)	− 0.06	0.915
HET density (ref: no)	1.11 (0.48–1.75)	0.11	0.743	1.48 (0.70–2.23)	0.40	0.158
Swirl sign (ref: no)	1.27 (0.48–2.07)	0.24	0.55	0.77 (− 0.23–1.78)	− 0.25	0.622
Black hole sign (ref: no)	1.16 (0.57–1.74)	0.15	0.622	0.98 (0.20–1.76)	− 0.02	0.968
Blend sign (ref: no)	0.63 (− 0.74–1.99)	− 0.47	0.502	1.23 (0.12–2.34)	0.21	0.714
Fluid sign (ref: no)	0.50 (− 0.85–1.84)	− 0.70	0.307	1.32 (0.19–2.45)	0.28	0.628
Hypodensities (ref: no)	2.06 (1.48–2.64)	0.72	0.015	1.76 (0.62–2.89)	0.56	0.158

*β*, beta regression coefficient; *CI*, confidence interval; *GCS*, Glasgow Coma Scale; *HET density*, heterogeneous density; *IVH*, intraventricular hemorrhage; *IRR shape*, irregular shape; *OR*, odds ratio; *P*, *p* value; *ref*, reference

## Discussion

**Fig. 2:**
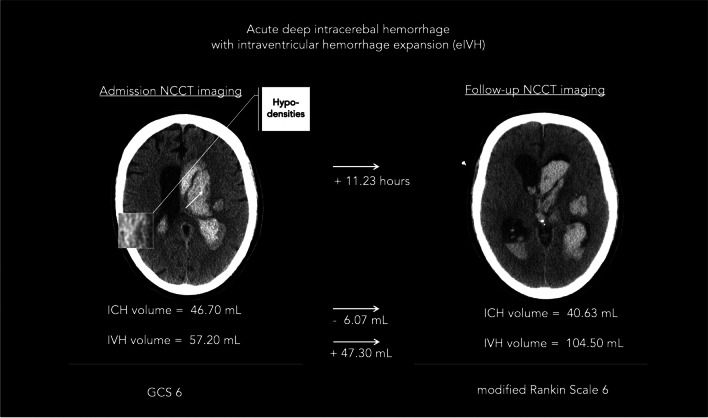
An illustrative example of non-contrast computed tomography (NCCT) markers associated with intraventricular hemorrhage expansion in patients with acute intracerebral hemorrhage. An illustrative example of a patient with intraventricular hemorrhage expansion (eIVH) defined as absolute volume increase of intraventricular hemorrhage (IVH) between admission (left side) and follow-up NCCT imaging (right side); given with the presence of hypodensities and corresponding volumes for intracerebral hemorrhage (ICH) and IVH, admission Glasgow Coma Scale (GCS), and clinical outcome at 90 days with modified Rankin Scale. ICH, intracerebral hemorrhage; GCS, Glasgow Coma Scale; IVH, intraventricular hemorrhage; mL, milliliters; NCCT, non-contrast computed tomography

**Fig. 3:**
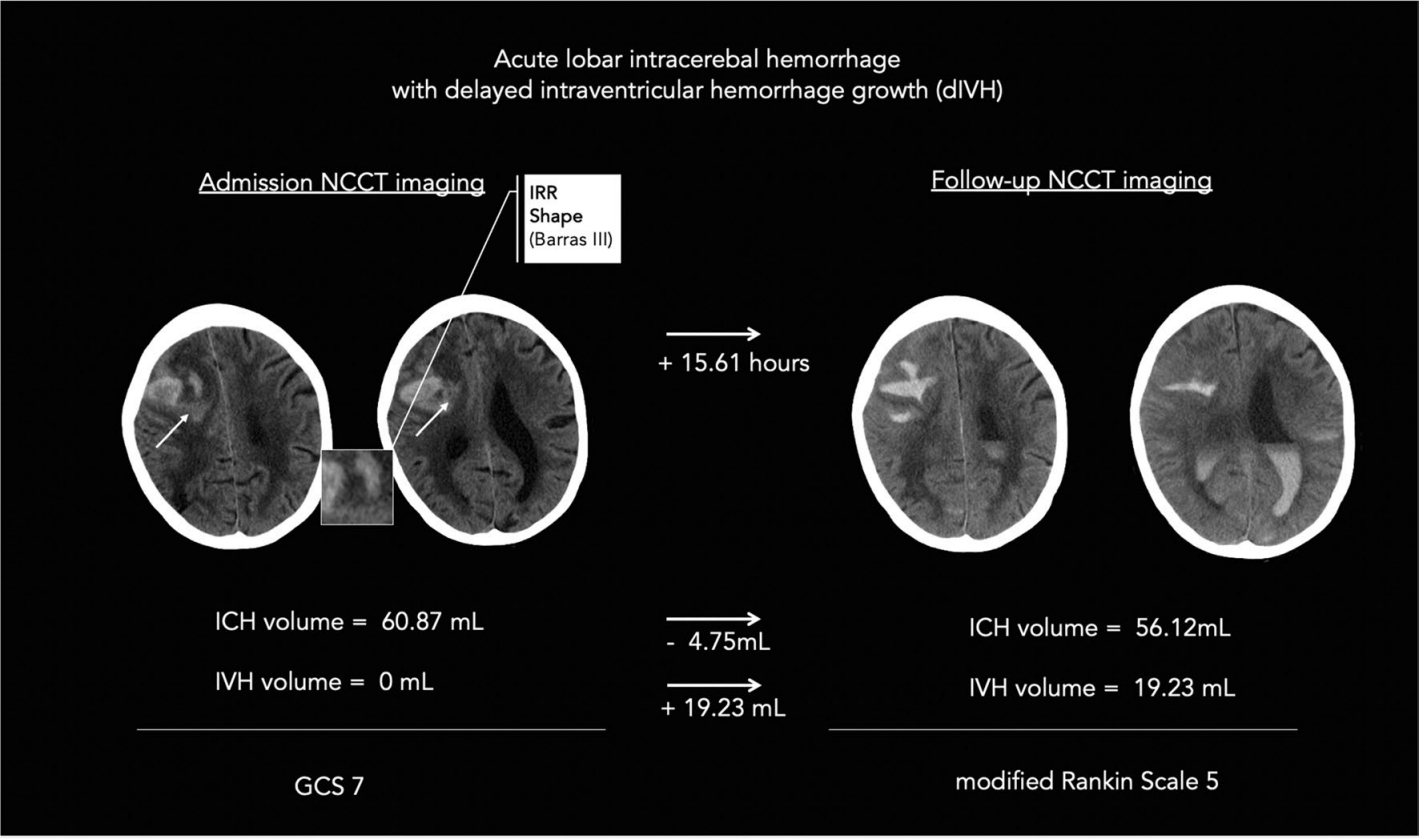
An illustrative example of non-contrast computed tomography (NCCT) markers associated with delayed intraventricular hemorrhage in patients with acute intracerebral hemorrhage. An illustrative example of a patient with delayed intraventricular hemorrhage (dIVH) defined as not present on admission imaging (left side) and any newly occurring IVH on follow-up NCCT imaging (right); given with the presence of irregular shape and corresponding volumes for intracerebral hemorrhage (ICH) and IVH, admission Glasgow Coma Scale (GCS), and clinical outcome at 90 days with modified Rankin Scale. ICH, intracerebral hemorrhage; GCS, Glasgow Coma Scale; IRR shape, irregular shape; IVH, intraventricular hemorrhage; mL, milliliters; NCCT, non-contrast computed tomography

In this study, we aimed to validate findings on the association of NCCT features with IVH growth and to determine subgroup-specific association with eIVH and dIVH in patients with acute spontaneous ICH. Our results confirmed that NCCT features were strong predictors of IVH growth [10]. In our subgroup analysis, eIVH was significantly associated with hypodensities whereas dIVH was significantly associated with irregular shape. Illustrative examples are given in Figures 2 and 3. Further, two important previous findings on the relationship of IVH growth and functional outcome were confirmed: Both eIVH and dIVH had a negative effect on the functional outcome as seen in our multivariate regression analysis (Supplementary Material Table 4) [7, 8]. Secondly, the revised HE definition had a significantly higher diagnostic accuracy in the prediction of poor functional outcome compared to the conventional definition of HE (Supplementary Table 5) [9, 10]. The pathophysiological mechanisms underlying these associations still remain unclear. Therefore, we hypothesized a mediation effect of parenchymal hematoma expansion, but our findings did not confirm this hypothesis, suggesting that the link between NCCT features and IVH growth is not a simple epiphenomenon of parenchymal bleeding [37]. Consistent with this finding, we also noted that most patients experiencing IVH growth did not have parenchymal hematoma expansion. We also explored a potential interaction with ICH location and time from onset to initial imaging but found no significant effect of these potential confounders on the association between NCCT features and increased risk of IVH growth [8, 33, 34]. From a clinical perspective, NCCT features may help clinicians in the stratification and early identification of patients at a high risk of neurological deterioration because of IVH growth. Recent results from the STOP-AUST trial indicate that the severity of IVH growth may be attenuated by tranexamic acid treatment following ICH [5]. Therefore, NCCT features may improve patients’ selection in future clinical trials, identifying subjects at a high risk of IVH growth and therefore more likely to benefit from hemostatic treatment. Some limitations of our analysis should be considered. First, our findings were derived from a retrospective analysis and require prospective validation. Second, the imaging protocol was not standardized across participating sites, although there is no evidence that the NCCT acquisition technique influences NCCT markers’ detection [16]. Third, imaging data were collected at two time points only and we are unable to assess potential dynamics of IVH volumes beyond this time frame. Fourth, NCCT markers were rated by well-experienced ICH researchers and may vary in raters with different levels of experience; however, results agreed well with our previous findings [15]. Finally, blood pressure control and coagulopathy reversal might have influenced the risk of IVH growth and were not accounted for. Finally, the described associations between NCCT markers and IVH growth may not necessarily imply causality, and further research is needed to characterize the underlying biological mechanisms.

## Conclusions

NCCT features independently predict IVH growth, and this association was independent of imaging time and ICH location and not mediated by parenchymal hematoma growth HE. Our findings suggest the possibility to stratify the risk of IVH growth with baseline NCCT and might inform ongoing and future studies.

### Supplementary Information

Below is the link to the electronic supplementary material.

Supplementary file1 (PDF 272 KB)

## Abbreviations

%: Percentage
CI: Confidence interval
CTA: Computed tomography angiography
DICOM: Digital Imaging and Communications in Medicine
dIVH: Delayed intraventricular hemorrhage
eIVH: Intraventricular hemorrhage expansion
EVD: External ventricular drain
GCS: Glasgow Coma Scale
HE: Hematoma expansion
HET density: Heterogeneous density
HU: Hounsfield units
ICC: Intraclass correlation coefficient
ICH: Intracerebral hemorrhage
IQR: Interquartile range
IRR shape: Irregular shape
IVH: Intraventricular hemorrhage
LSW: Last seen well
mRS: Modified Rankin Scale
NCCT: Non-contrast computed tomography
NifTI: Neuroimaging Informatics Technology Initiative
OR: Odds ratio
PACS: Picture archiving and communication system
ROI: Regions of interest
SD: Standard deviation
VIF: Variance inflation factor

## Declarations

### Guarantor

The scientific guarantor of this publication is Dr. Andrea Morotti.

### Statistics and biometry

One of the authors has significant statistical expertise.

### Study subjects or cohorts overlap

Some study subjects or cohorts have been previously reported in Nawabi J, et al Imaging-based outcome prediction of acute intracerebral hemorrhage. *Transl Stroke Res*. 2021 Dec;12(6):958–967. https://doi.org/10.1007/s12975-021-00891-8. Epub 2021 Feb 6.

### Methodology

• retrospective

• observational

• multicenter study
