# Correction to: Non-contrast computed tomography features predict intraventricular hemorrhage growth

**DOI:** 10.1007/s00330-023-09896-3

**Published:** 2023-07-19

**Authors:** Jawed Nawabi, Frieder Schlunk, Andrea Dell’Orco, Sarah Elsayed, Federico Mazzacane, Dmitriy Desser, Ly Vu, Estelle Vogt, Haoyin Cao, Maik F. H. Böhmer, Burak Han Akkurt, Peter B. Sporns, Marco Pasi, Ulf Jensen-Kondering, Gabriel Broocks, Tobias Penzkofer, Jens Fiehler, Alessandro Padovani, Uta Hanning, Andrea Morotti

**Affiliations:** 1 Department of Radiology, Charité - Universitätsmedizin Berlin, Campus Mitte, Humboldt-Universität Zu Berlin, Freie Universität Berlin, Berlin Institute of Health, Charitéplatz 1, 10117 Berlin, Germany; 2 Berlin Institute of Health (BIH), BIH Biomedical Innovation Academy, Berlin, Germany; 3 Department of Neuroradiology (CCM), Charité - Universitätsmedizin Berlin, Campus Mitte, Humboldt-Universität Zu Berlin, Freie Universität Berlin, Berlin Institute of Health, Berlin, Germany; 4 Department of Diagnostic and Interventional Neuroradiology, University Medical Center Hamburg Eppendorf, Hamburg, Germany; 5 Department of Brain and Behavioral Sciences, University of Pavia, Pavia, Italy; 6 U.C. Malattie Cerebrovascolari E Stroke Unit, IRCCS Fondazione Mondino, Pavia, Italy; 7 Department of Radiology, University Hospital Muenster, Muenster, Germany; 8 Department of Neuroradiology, Clinic for Radiology and Nuclear Medicine, University Hospital Basel, Basel, Switzerland; 9 Department of Neurology, University Hospital of Tours, Tours, France; 10 Department of Neuroradiology, University Hospital Schleswig-Holstein, Campus Lübeck, Lübeck, Germany; 11 Department of Radiology, Charité - Universitätsmedizin Berlin, Campus Virchow Klinikum, Humboldt-Universität Zu Berlin, Freie Universität Berlin, Berlin Institute of Health, Berlin, Germany; 12 Department of Clinical and Experimental Sciences, Neurology Clinic, University of Brescia, Brescia, Italy; 13 Neurology Unit, Department of Neurological Sciences and Vision, ASST-Spedali Civili, Brescia, Italy

**Correction to: European Radiology**

https://doi.org/10.1007/s00330-023-09707-9

In this article, the affiliation details for author Ulf Jensen-Kondering were incorrectly given as:

Department of Radiology and Neuroradiology, University

Hospital Schleswig–Holstein, Campus Kiel, Kiel, Germany

The correct affiliation should have been:

Department of Neuroradiology, University Hospital Schleswig–Holstein, Campus Lübeck, Lübeck, Germany

Additionally, the author name Andrea Dell’Orco was given incorrectly as Andrea Dell Orco. The original article has been corrected.

