# Supplementary material for: Non-contrast computed tomography features predict intraventricular hemorrhage growth
Source: Eur Radiol. 2023 May 22;33(11):7807–17. doi: 10.1007/s00330-023-09707-9 (PMC10598100; doi:10.1007/s00330-023-09707-9)
Supplement: Supplementary file 1 — Supplementary file1 (PDF 272 KB) [file 330_2023_9707_MOESM1_ESM.pdf]

ELECTRONIC SUPPLEMENTARY MATERIAL

Non-contrast Computed Tomography Features Predict Intraventricular Hemorrhage Growth

Table 1: Interrater agreement of two raters for Noncontrast Computed Tomography (NCCT) signs stratified across one reading.

Rater 1	Rater 2	Cohen's kappa*	95% CI	SE	P-value
Shape Markers					
IRR Shape	IRR Shape	0.88	0.85-0.92	0.018	<0.001
Satellite Sign	Satellite Sign	0.75	0.68-0.82	0.036	<0.001
Island Sign	Island Sign	0.82	0.74-0.91	0.043	<0.001
Density Markers					
HET Density	HET Density	0.85	0.80-0.90	0.024	<0.001
Swirl Sign	Swirl Sign	0.74	0.67-0.80	0.033	<0.001
Black Hole Sign	Black Hole Sign	0.76	0.68-0.85	0.043	<0.001
Blend Sign	Blend Sign	0.70	0.58-0.82	0.063	<0.001
Fluid Sign	Fluid Sign	0.95	0.89-1.00	0.029	<0.001
Hypodensities	Hypodensities	0.84	0.80-0.88	0.020	<0.001

Legend: Interrater agreement of nine Noncontrast Computed Tomography Markers specified with kappa with 95% confidence interval (CI) and standard error (SE). *calculated cohen's kappa across two raters in one rating for n=731 subjects.

Table 2: Intrarater agreement of one rater for Noncontrast Computed Tomography (NCCT) signs stratified across two readings.

Rater 1	Rater 1	Cohen's kappa*	95% CI	SE	P-value
Shape Markers					
IRR Shape	IRR Shape	0.87	0.83-0.91	0.019	<0.001
Satellite Sign	Satellite Sign	0.93	0.88-0.97	0.023	<0.001
Island Sign	Island Sign	0.95	0.90-1.00	0.025	<0.001
Density Markers					
HET Density	HET Density	0.80	0.76-0.83	0.018	<0.001
Swirl Sign	Swirl Sign	0.94	0.91-0.98	0.018	<0.001
Black Hole Sign	Black Hole Sign	0.98	0.96-1.01	0.013	<0.001
Blend Sign	Blend Sign	0.96	0.90-1.02	0.030	<0.001
Fluid Sign	Fluid Sign	0.95	0.90-1.00	0.024	<0.001
Hypodensities	Hypodensities	0.81	0.77-0.86	0.021	<0.001

Legend: Intrarater agreement of nine Noncontrast Computed Tomography Markers specified with kappa with 95% confidence interval (CI) and standard error (SE). *calculated cohen's kappa across one rater in two ratings for n=100 subjects.

Table 3: Intraclass Correlation of one rater for semimanual volume segmentation of intracerebral hemorrhage and intraventricular hemorrhage stratified across two readings.

**Region of interest**	**Intraclass Correlation***	**95% Lower CI**	**95% Upper CI**	***P*-Value**
Intracerebral hemorrhage (ICH; n= 100)	0.9973	0.9955	0.9983	<0.001
Intraventricular hemorrhage (IVH; n= 100)	0.9946	0.9918	0.9964	<0.001

Legend: Intraclass agreement across hundred randomly selected and quantified volumes of intracerebral hemorrhage and intraventricular hemorrhage on computed tomography specified with intraclass correlation (ICC) with 95% confidence interval (CI). *Stratified ICC across one rater in two ratings.

Table 4: Multivariate logistic regression analysis on the analysis of predictors of poor functional outcome.

Poor functional outcome*	OR (95% CI)	β	*P*-Value
Hypertension [ref: no]	1.76 (1.05-2.96)	0.57	0.032
Admission GCS	0.79 (0.73-0.84)	-0.239	<0.001
Expanded IVH [ref: no]	2.14 (1.11-4.15)	0.76	0.024
Delayed IVH [ref: no]	2.13 (1.03-4.42)	0.76	0.043

Legend: β indicates beta regression coefficient; ICH, intracerebral hemorrhage volume; IVH, intraventricular hemorrhage; OR, odds ratio; *P*, p-value; ref, reference. *Adjusted for age, sex, use of anticoagulation, baseline ICH volume, and ICH location.

Table 5: Receiver operating characteristic for three different markers of active bleeding in the prediction of clinical outcome and mortality with pairwise comparison.

**Clinical Outcome**	**AUC**	**95% CI**	**SE**	**Sensitivity**	**Specificity**	***PPV***	***NPV***	***P*-Value**
HE	0.574	0.541-0.606	0.011	21.03	92.24	88.52	29.10	<0.0001
Revised HE	0.602	0.570-0.634	0.017	42.03	78.43	84.72	32.22	<0.0001
IVH Growth	0.587	0.554-0.619	0.013	27.35	88.36	86.99	29.94	<0.0001

Pairwise AUC comparison	Δ AUC	95% CI	SE	*P*-Value*
IVH growth ~revised HE	0.016	-0.007-0.038	0.011	0.177
IVH growth ~ HE	0.013	-0.024-0.051	0.019	0.488
Revised HE ~ HE	0.029	0.002-0.056	0.014	0.034

**Mortality**	**AUC**	**95% CI**	**SE**	**Sensitivity**	**Specificity**	***PPV***	***NPV***	***P*-Value**
HE	0.593	0.561-0.624	0.014	27.54	86.01	38.33	78.99	<0.0001
Revised HE	0.611	0.579-0.642	0.018	53.25	68.98	35.15	82.37	<0.0001
IVH Growth	0.604	0.572-0.635	0.018	33.44	80.78	35.13	82.36	<0.0001

Pairwise AUC comparison	Δ AUC	95% CI	SE	*P*-Value*
IVH growth ~revised HE	0.007	-0.016-0.038	0.012	0.176
IVH growth ~ HE	0.011	-0.029-0.051	0.021	0.592
Revised HE ~ HE	0.018	-0.111-0.048	0.012	0.539

* *P*-value of the test of DeLong et al. (1988)

Legend: Receiver operating characteristic curves for intraventricular hemorrhage (IVH) growth, hematoma expansion (HE) and revised hematoma expansion (rHE) and pairwise comparison of ROC analysis with difference in area under the curve (Δ AUC) for the prediction of clinical outcome **(above)** and mortality **(below)**. AUC indicates area under the curve; 95% CI, confidence interval; IVH, intraventricular hemorrhage expansion; HE, hematoma expansion; NPV, negative predictive value; PPV, positive predictive value; SE, standard error.

Figure 1: Illustrative examples Noncontrast Computed Tomography (NCCT) Markers in patients with intracerebral hemorrhage.

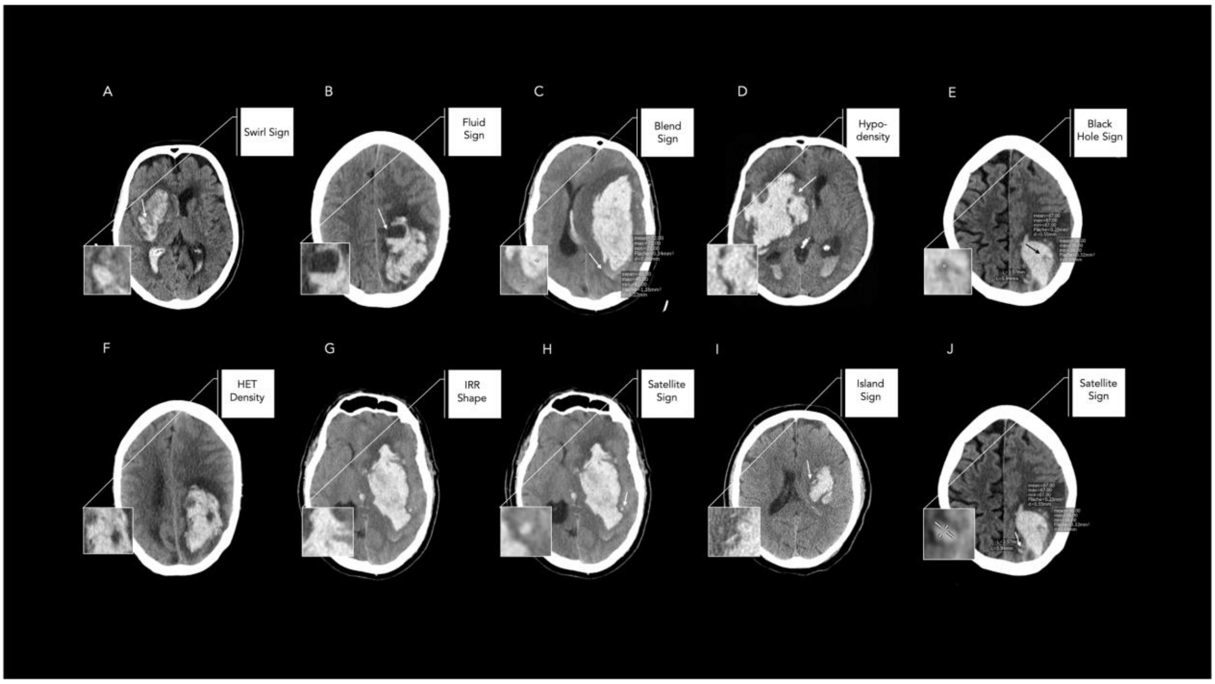

Legend: Illustrative examples of nine noncontrast Computed Tomography (NCCT) markers of hemorrhage expansion. Axial slices of admission NCCTs in acute intracerebral hemorrhage (ICH) with swirl sign (A), fluid sign (B), blend sign (C), hypodensity also qualifying as swirl sign, (E), black hole sign (F) also qualifying as swirl sign and hypodensity, heterogenous density (F), irregular shape (G), satellite sign (H and J), and island sign (I). IRR Shape; indicates irregular shape; HET Density, heterogenous density.

Moderator and Mediator Analysis

M: Mediator - Indirect effect of X on Y through M
X: Independent Variable
Y: Outcome
W: Moderator 1 - Conditional direct effect of X on Y
Z: Moderator 2 - Conditional direct effect of X on Y

1. IVH Growth

1.1. Mediator Analysis – Model Nr. 4

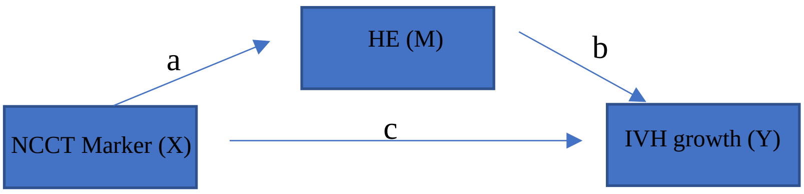

- The **"a" path** represents the effect on HE and the **"b" path** the effect of HE on IVH growth (indirect effect)
- The **"b" path** represents the effect of HE on IVH growth
- The **„c" path** represents the direct effect of IRR Shape on IVH growth (direct effect)

Path a: For the first model, IRR shape emerged as a positive and but not significant predictor of HE (beta= 89.9091, SE= 163.2859, p= 0.5829).

Path b: The direct effect of HE on IVH growth was positive and but not statistically significant (beta= 0.0004, SE= 0.0006, p-value= 0.5046).

We tested this indirect effect (path a*b) for statistical significance by examining the bootstrap confidence interval provided. If the null of 0 falls between the lower and upper bound of the interval, we concluded that the indirect effect is not significant. If the null of 0 falls outside of the interval, we rejected the null and concluded we had a significant indirect effect.

The bootstrap confidence interval from our data [lower limit -0.0769, upper limit 1.8090] does contain 0, so we conclude that the indirect effect was not statistically significant.

Path c: IRR shape was a significant positive predictor of IVH growth (beta= 1.2087, SE= 0.0517, p-value = 0.016).

Note: The Model was adjusted for covariates as presented in the multivariate regression analysis.

1.2. Moderator Analysis - Model Nr. 2

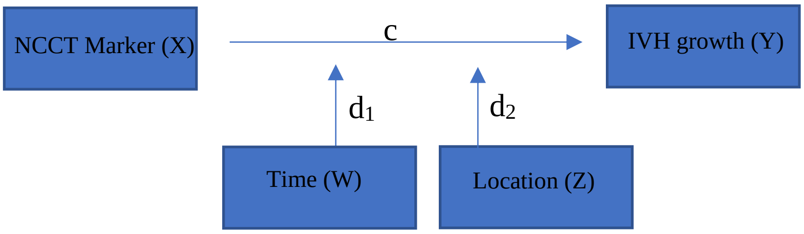

- The **„c“ path** represents the direct effect of IRR Shape on IVH growth (direct effect)
- The **“d” path** shown here represents effect of HE on the slope for path “c” (interaction effect)

Path c: IRR shape was still a positive and significant predictor of IVH growth (beta= 2.3693, SE= 0.9267, p-value= 0.016).

Path d: The missing statistical significance for the interaction term 1 (Int_1: IRR Shape x time; beta= -0.0155, SE= 0.0316, p-value= 0.6230) demonstrated that the path from IRR shape to IVH growth was not moderated by time from symptom onset/LSW to imaging. Also, the missing statistical significance for the interaction term 2 (Int_2: IRR Shape x location; beta= -1.4845, SE= 1.13, p-value= 0.1890) demonstrated that the path from IRR shape to IVH growth was not moderated by hematoma location.

Note: The Model was adjusted for covariates as presented in the multivariate regression analysis; except for location as it was tested as a moderator.

Conclusion: IRR shape was a positive predictor of IVH growth. The effect was not mediated by HE. There was no interaction found between IRR shape and IVH growth with the time from symptom onset/LSW to imaging or hematoma location.

2. Subgroup analysis

<u>2.1 IVH expansion (eIVH)</u>

2.1.1. Mediator Analysis – Model Nr. 4

Path a: For the first model, IRR shape emerged as a negative and not significant predictor of HE (beta= -133.4556, SE= 428,7929, p= 0.7568).

Path b: The direct effect of HE one eIVH was positive and not statistically significant (beta= -0.0002, SE= 0.0005, p-value= 0.7547).

We tested this indirect effect (path a*b) for statistical significance by examining the bootstrap confidence interval provided. If the null of 0 falls between the lower and upper bound of the interval, we concluded that the indirect effect is not significant. If the null of 0 falls outside of the interval, we rejected the null and concluded we had a significant indirect effect.

The bootstrap confidence interval from our data [lower limit -1.4914, upper limit 1.0043] did contain 0, so we concluded that the indirect effect was not statistically significant.

Path c: IRR shape was a positive and but not a significant predictor of eIVH (beta= 0.9132, SE= 0.9493, p-value = 0.3360).

Note: The Model was adjusted for covariates as presented in the multivariate regression analysis.

2.2.2. Moderator Analysis - Model Nr. 2

Path c: IRR shape was a positive but not significant predictor of IVH growth (beta= 0.3995, SE= 0.5985, p-value= 0.5045).

Path d: The missing statistical significance for the interaction term 1 (Int_1: IRR Shape x location; beta= 0.0094, SE= 0.0273, p-value= 0.7305) demonstrated that the path from IRR shape to eIVH was not moderated by hematoma location. Also, the missing statistical significance for the interaction term 2 (Int_2: IRR Shape x time; beta= -0.4991, SE= 0.7747, p-value= 0.5194) demonstrated that the path from IRR shape to eIVH was not moderated by time.

Note: The Model was adjusted for covariates as presented in the multivariate regression analysis; except for location as it was tested as a moderator.

Conclusion: IRR shape was a positive but not significant direct predictor of eIVH. The effect was not mediated by HE. There was no interaction found between IRR shape and eIVH expansion with the time from symptom onset/LSW to imaging.

<u>2.2 delayed IVH (dIVH)</u>

2.2.1. Mediator Analysis – Model Nr. 4

Path a: For the first model, hypodensities emerged as a negative and not significant predictor of HE (beta= -420.1195, SE= 296.5059, p= 0.1623).

Path b: The direct effect of HE one dIVH was negative and was not statistically significant (beta= -0.0001, SE= 0.0006, p-value= 0.8396).

We tested this indirect effect (path a*b) for statistical significance by examining the bootstrap confidence interval provided. If the null of 0 falls between the lower and upper bound of the interval, we concluded that the indirect effect is not significant. If the null of 0 falls outside of the interval, we rejected the null and concluded we had a significant indirect effect.

The bootstrap confidence interval from our data [lower limit -0.1818, upper limit 1.7206] did contain 0, so we concluded that the indirect effect is not statistically significant.

Path c: Hypodensities was a positive but not significant predictor of dIVH (beta= 0.9241, SE= 0.6449, p-value = 0.1519).

Note: The Model was adjusted for covariates as presented in the multivariate regression analysis.

2.2.2. Moderator Analysis - Model Nr. 2

Path c: Hypodensities was positive but not a significant positive predictor of dIVH (beta= 0.0.2138, SE= 0.5677, p-value= 0.7064).

Path d: The missing statistical significance for the interaction term 1 (Int_1: Hypodensities x location; beta= 0.9880, SE= 0.8197, p-value= 0.0.2281) demonstrated that the path from Hypodensities to dIVH was not moderated by hematoma location. Also, the missing statistical significance for the interaction term 2 (Int_2: Hypodensities x time; beta=-0.071, SE= 0.0418, p-value= 0.892) demonstrated that the path from Hypodensities to dIVH was not moderated by time.

Note: The Model was adjusted for covariates as presented in the multivariate regression analysis; except for location as it was tested as a moderator.

Conclusion: Hypodensities was a positive but not significant direct predictor of IVH growth. The effect was not mediated by HE. There was no interaction found between Hypodensities and dIVH with the time from symptom onset/LSW to imaging.
